# Phenotype-oriented network analysis for discovering pharmacological effects of natural compounds

**DOI:** 10.1038/s41598-018-30138-w

**Published:** 2018-08-03

**Authors:** Sunyong Yoo, Hojung Nam, Doheon Lee

**Affiliations:** 1 Department of Bio and Brain Engineering, Korea Advanced Institute of Science and Technology (KAIST), Daejeon, 34141 Republic of Korea; 2 Bio-Synergy Research Center, Daejeon, 34141 Republic of Korea; 3 School of Electrical Engineering and Computer Science, Gwangju Institute of Science and Technology (GIST), Gwangju, 61005 Republic of Korea

## Abstract

Although natural compounds have provided a wealth of leads and clues in drug development, the process of identifying their pharmacological effects is still a challenging task. Over the last decade, many *in vitro* screening methods have been developed to identify the pharmacological effects of natural compounds, but they are still costly processes with low productivity. Therefore, *in silico* methods, primarily based on molecular information, have been proposed. However, large-scale analysis is rarely considered, since many natural compounds do not have molecular structure and target protein information. Empirical knowledge of medicinal plants can be used as a key resource to solve the problem, but this information is not fully exploited and is used only as a preliminary tool for selecting plants for specific diseases. Here, we introduce a novel method to identify pharmacological effects of natural compounds from herbal medicine based on phenotype-oriented network analysis. In this study, medicinal plants with similar efficacy were clustered by investigating hierarchical relationships between the known efficacy of plants and 5,021 phenotypes in the phenotypic network. We then discovered significantly enriched natural compounds in each plant cluster and mapped the averaged pharmacological effects of the plant cluster to the natural compounds. This approach allows us to predict unexpected effects of natural compounds that have not been found by molecular analysis. When applied to verified medicinal compounds, our method successfully identified their pharmacological effects with high specificity and sensitivity.

## Introduction

Natural compounds and their derivatives have been used as a valuable source of medicinal agents. To date, an impressive number of modern drugs have been derived from natural sources, many based on their use in herbal medicine^1–3^. Herbal medicine has accumulated considerable knowledge about the medicinal use of plants over the last thousand years. Additionally, herbal medicine is presumed to be safe, harmless and without side effects because of its natural origins^4,5^. Recent surveys showed that approximately 70–80% of the world’s population depends on herbal medicine for their primary health care^6,7^. However, only a small number of plant species have been investigated by scientists and approved for commercial purposes while more than 35,000 plant species are used for medicinal purposes worldwide^8,9^. Therefore, a better understanding of herbal medicine through scientific analysis will provide new insights for drug development.

Most previous studies on finding medicinal agents from herbal medicine were performed by *in vitro* assessment. The plant associated with the disease of interest was selected from herbal medicine. Then, the natural compound or plant itself was extracted, and its biological activities were confirmed by *in vitro* screening methods^10–13^. However, large-scale experiments are required to analyze a large number of constituent natural compounds, and the problem increases exponentially as the number of plants under consideration increases. Therefore, *in silico* approaches, such as similarity-based, network-based or mechanism-based methods, have been proposed to filter potential medicinal agents from numerous natural compounds^14–17^. Most of these studies have used herbal medicine information only as a preliminary tool to select plants or natural compounds for a certain disease. They focused on molecular analysis, such as molecular structure or target protein similarity, to predict the potential effects of natural compounds. However, many natural compounds do not have molecular structural information available, and their target protein information remains mostly unknown (Supplementary Fig. 1). Hence, these approaches often encounter obstacles to large-scale analysis^18–20^.

The accumulated knowledge of herbal medicine, especially information on the efficacy of medicinal plants, can be used as a key resource to overcome this limitation. Even if no molecular information on many natural compounds is available, large-scale analysis can be performed by investigating the relationship between the known efficacy of plants and natural compounds from information on herbal medicine. For this purpose, we should consider the following characteristics of herbal medicine: (i) The efficacy of medicinal plants is described in various phenotype terms (Supplementary Fig. 2). The efficacy information contains both high-level concepts, such as inflammation and hormone imbalance, and low-level concepts, such as aortitis and diabetic retinopathy (Supplementary Fig. 3). Furthermore, similar concepts, such as synonyms and symptoms of diseases, are described in various forms. Therefore, to utilize the information on plant efficacy, these complex associations should be considered. For example, when extracting plants associated with urination, we can achieve more relevant results by examining phenotypes associated with urination, such as dysuria, urethral stones, and urinary tract abnormalities. (ii) Medicinal plants contain numerous natural compounds^21^. Unlike a single-target drug, herbal medicines consist of complex multicomponent mixtures of natural compounds. Moreover, even if we select plants that are associated with a particular disease, they are likely to be associated with many other diseases. Therefore, analyzing which natural compound in the plant is associated with a particular disease is difficult.

Here, we present a phenotype-oriented network analysis to identify pharmacological effects of natural compounds from herbal medicine. To address the characteristics of plant efficacy information in herbal medicine, the relationships between known plant efficacy and 5,021 phenotypes were quantified by applying a random walk with restart (RWR) algorithm, taking into account the hierarchy of the phenotypic network. This approach allows us to extract plant clusters with similar efficacy by considering complex phenotype associations. We then hypothesized that significantly enriched natural compounds in a plant cluster would be closely related to the efficacy associated with the plant cluster. To test this hypothesis, we investigated the predicted pharmacological effects of natural compounds based on the verified and candidate effect sets. We found that our predictions covered a large number of the results reported in previous work. More importantly, this approach solved the bottleneck by predicting pharmacological effects of natural compounds that were difficult to analyze due to a lack of molecular information. In conclusion, the novelty of our method is threefold: (i) It is the first phenotype-based *in silico* method that identifies pharmacological effects of natural compounds from herbal medicine without molecular analysis. (ii) Large-scale analysis can be performed by addressing the characteristics of herbal medicine systematically. (iii) It can be used as a preliminary tool to screen medicinal agents from numerous natural compounds.

## Materials and Methods

### Phenotype-oriented network analysis

**Figure 1:**
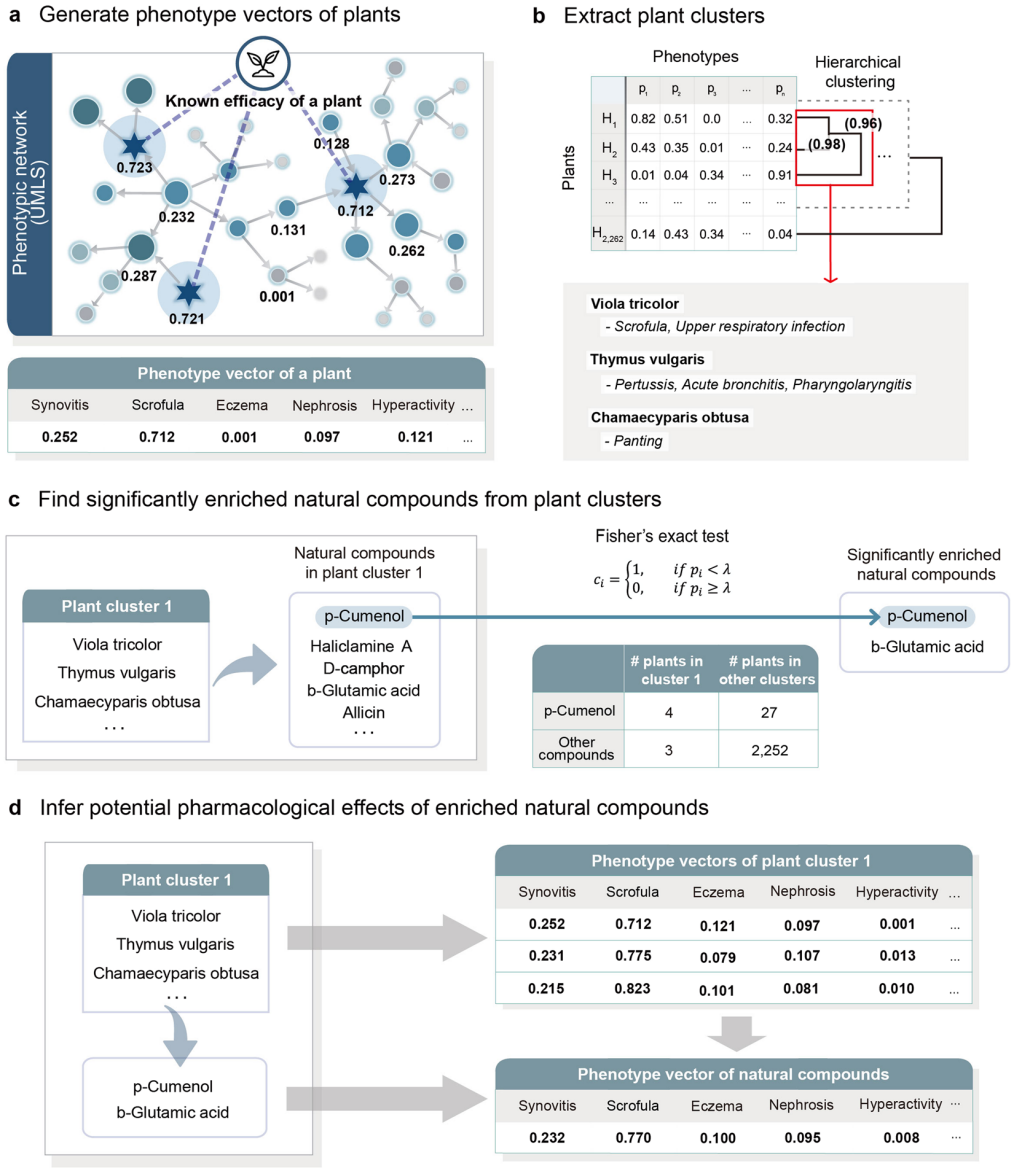
A systematic overview of the phenotype-oriented network analysis. (**a**) Phenotype values of a plant were obtained by calculating the quantified relationship between phenotypes on the phenotypic network. In the phenotypic network, the RWR algorithm was performed based on the known efficacy of the plant (star), and the RWR results are shown as colored nodes. The phenotype vector of a plant was constructed based on the RWR results. (**b**) Plants with similar pharmacological effects were grouped by applying hierarchical clustering analysis to the matrix of phenotype vectors. Hierarchical clustering was performed by using the pvclust. The approximately unbiased *p*-values (bracketed values) calculated for each branch in the clustering represent the support in the data for the observed subtree clustering. Clusters with *p*-value over 0.95 (red box) are strongly supported by the data. (**c**) All natural compounds contained in the plant cluster were extracted. For each natural compound (*c*_*i*_), Fisher’s exact test was performed to check whether the natural compound was significantly enriched in the cluster. Finally, natural compounds with *p*-values (*p*_*i*_) of Fisher’s exact test lower than a threshold value (λ) were selected. (**d**) The pharmacological effects of an enriched natural compound were obtained by mapping the averaged phenotype vectors of the plant cluster enriched this specific natural compound.

We designed a novel algorithm to identify pharmacological effects of natural compounds from herbal medicine. The algorithm consists of four steps (Fig. 1): (i) constructing phenotype vectors of plants by investigating the relationships between known plant efficacy and thousands of phenotypes in a phenotypic network; (ii) extracting plant clusters with similar efficacy by applying hierarchical clustering to phenotype vectors; (iii) finding significantly enriched natural compounds from the plant clusters; and (iv) identifying potential pharmacological effects of the natural compounds.

We generated phenotype vectors that cover a large number of quantified pharmacological effects of plants (Fig. 1a). Each phenotype vector contains 5,021 phenotypes defined by Medical Subject Headings (MeSH) and Online Mendelian Inheritance in Man (OMIM) (Supplementary Data 1). In the phenotypic network, phenotype nodes close to the root node have broad concepts, phenotype nodes distant from the root node have narrow concepts. Therefore, we assigned the edge weight between nodes in the phenotype network based on semantic similarity, which measures how similar two phenotypes are by determining closeness in a hierarchy. Next, the pharmacological effects of the plants were quantified by applying the RWR algorithm to the edge-weighted phenotypic network. The initial values of the phenotypic network were assigned to the known efficacy of plants, and their diffusion states were calculated by the RWR algorithm. In this process, we generated phenotype vectors for 2,286 plants.

Next, hierarchical clustering was performed to extract plant clusters from the phenotype vectors (Fig. 1b). In contrast to previous studies that selected plants with a specific phenotype, we extracted plant clusters with similar efficacy by taking into account a large number of phenotypes with their hierarchical relationships. For example, *Viola tricolor*, *Thymus vulgaris* and *Chamaecyparis obtusa* have been clustered because they are known to be effective in respiratory-related diseases or symptoms, such as scrofula, pertussis, and panting. Each plant cluster consists of an average of 3.6 plants containing an average of 43.3 natural compounds. Since each plant cluster contains a large number of natural compounds, the relationship between the pharmacological effects of the plant cluster and the natural compounds is complex. To solve this problem, we extracted significantly enriched natural compounds from plant clusters by using Fisher’s exact test (Fig. 1c) and selecting natural compounds with a *p*-value lower than a threshold value. Finally, we investigated the potential pharmacological effects of the natural compounds (Fig. 1d). Our underlying hypothesis was that statistically significant natural compounds present in a plant cluster would have the pharmacological effects of the plant cluster. Therefore, the phenotype vectors of the plants belonging to the plant cluster were averaged and mapped to the enriched natural compounds.

### Data collection

Plant and natural compound information was collected from KTKP (http://www.koreantk.com/ktkp2014/), TCMID^22^, TCMSP^16^, TCM@Taiwan^23^, TCM-ID^24^ and KAMPO (http://kampo.ca/), covering Korean, Chinese and Japanese herbal medicine. The phenotypic network was taken from the 2017AA version of the Unified Medical Language System (UMLS)^25^, which provides integrated information on various terminologies related to biomedicine. The Metathesaurus is the main component of the UMLS and is organized by biomedical concepts, where each distinct concept is assigned a concept unique identifier (CUI). We collected CUIs with broader (RB) and narrower (RN) relationships from the MRREL lists, resulting in a total of 786,002 CUIs and 2,487,620 relations (Supplementary Data 2).

To validate the proposed method, we collected the following information. Drug information was acquired from DrugBank v.4.0^26^. Potential effects of natural compounds were collected from CTD^27^ and ClinicalTrials.gov^28^. Compound-gene associations were collected from DrugBank^26^, DCDB v.2.0^29^, CTD^27^, TTD^30^, BindingDB^31^, MATADOR^32^ and STITCH^33^. Gene-phenotype associations were collected from CTD^27^, DisGeNET^34^ and OMIM^35^. We also obtained protein-protein interaction (PPI) network data from BioGrid v.3.0.136^36^ and CODA v.1.0^37^.

### Quantifying the pharmacological effects of medicinal plants

**Figure 2:**
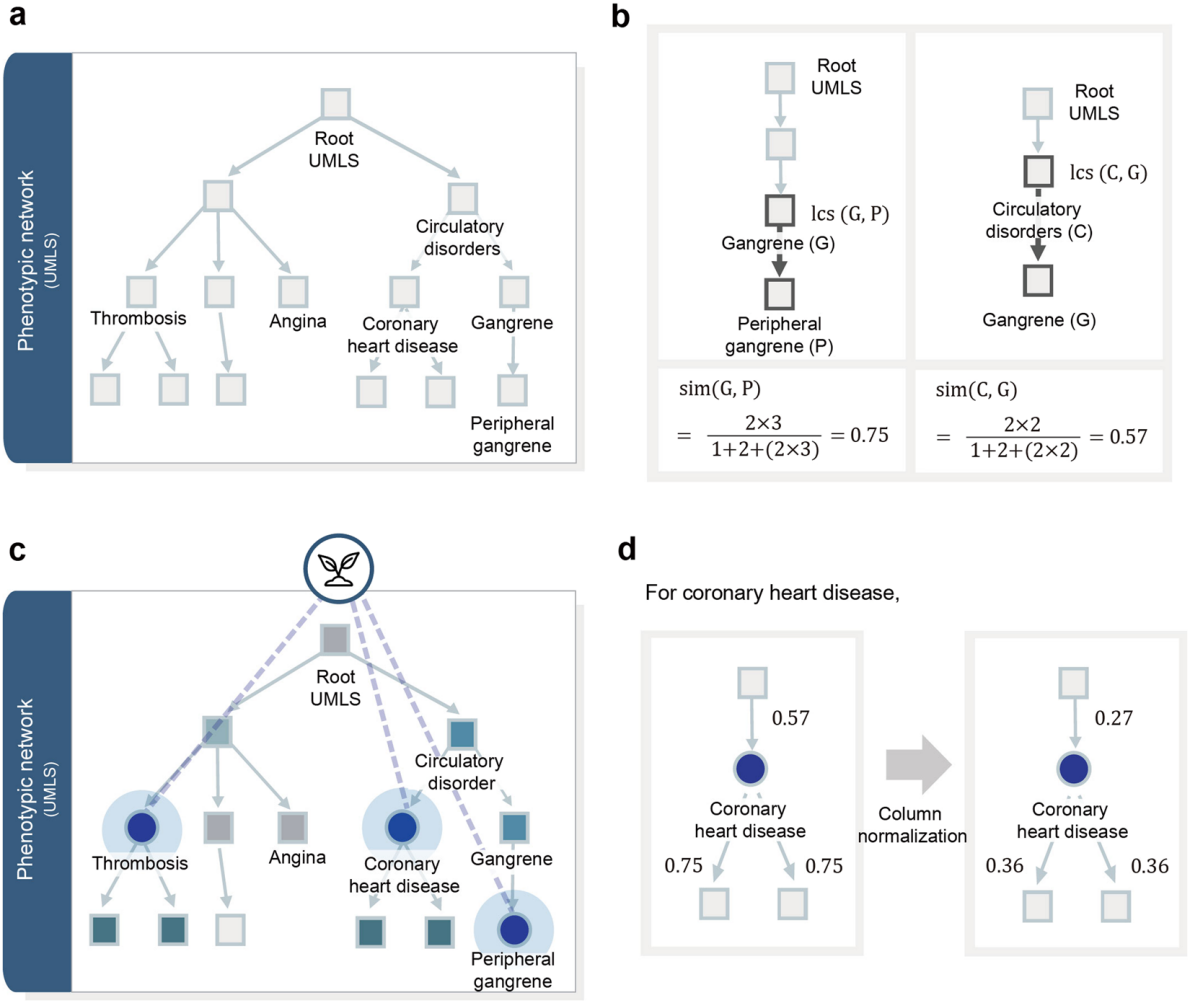
Quantifying the pharmacological effects of medicinal plants in the phenotypic network. (**a**) A phenotypic network was constructed based on the UMLS hierarchical relationships. (**b**) A semantic similarity between two phenotype concepts was calculated by considering the depth of the phenotypes and the distance between phenotypes. (**c**) In the phenotypic network, the RWR algorithm was performed based on the known efficacy of the plant (circle), and the RWR results are shown as colored nodes. (**d**) A transition matrix (*W*) is generated by the column normalization of the adjacency matrix based on the edge weights.

We constructed a phenotypic network based on the hierarchical relationship of UMLS^25^ and then calculated semantic similarity to measure the quantitative distance between phenotypes (Fig. 2a).

A relation between two general phenotype concepts, such as inflammation and hormonal imbalance, implies a reasonably large difference, while one between two specific concepts, such as diabetes mellitus and diabetic retinopathy, represents a small difference. Therefore, we applied the semantic similarity measure proposed by Wu & Palmer (wup)^38^ and defined by the following equation (Fig. 2b).

1 $${\rm{sim}}({c}_{1},{c}_{2})=\frac{2\times depth(lcs({c}_{1},{c}_{2}))}{path({c}_{1},lcs({c}_{1},{c}_{2}))+path({c}_{2},lcs({c}_{1},{c}_{2}))+2\times depth(lcs({c}_{1},{c}_{2}))}$$

where *lcs*(*c*_1_, *c*_2_) is the lowest common subsumer of concepts *c*_1_ and *c*_2_. We assigned the edge weights of the phenotypic network based on the semantic similarity scores between phenotype nodes. Next, we performed the RWR algorithm to investigate the quantified pharmacological effects of medicinal plants in the edge-weighted phenotypic network. RWR simulates a random walker from its seed nodes and iteratively transmits the node values to the neighbor nodes with probabilities proportional to the corresponding edge weights^39–41^. First, we assigned initial values to seed nodes in the phenotypic network based on the known efficacy information of a plant (Fig. 2c). Second, we calculated the transition probability from a node to its neighbor nodes. We assumed the transition probability to be the value of the quantified relationship between phenotypes on the phenotypic network. The transition probability vector of each node at time step *t* + 1 was defined as

2 $${p}_{t+1}=(1-r){W}^{T}{p}_{t}+r{p}_{0}$$

where *r* represents the restarting probability of the random walker at each time step, which we set to 0.7 in this study. *W* denotes a transition matrix that is the column normalization of the adjacency matrix based on the edge weight of the phenotypic network^42^ (Fig. 2d). *p*_*t*_ represents the probability vector of each node at time step *t*, and *p*_0_ represents the initial probability vector. The RWR algorithm simulates the random walker until all nodes reach the steady state (*p*_*t*+1_ − *p*_*t*_ < 10^−8^). We defined a list of phenotype values of a plant as a phenotype vector.

### Clustering plants based on phenotype similarity

We merged all phenotype vectors into a matrix and applied hierarchical clustering to extract plants with similar pharmacological effects. Hierarchical clustering was performed by using R module pvclust^43^, which involves multiscale bootstrap resampling of 1,000 iterations to assess statistical significance. We selected clusters with an approximately unbiased (AU) *p*-value greater than a specific threshold. The AU *p*-value indicates the extent to which a cluster is strongly supported by the data, and a higher AU *p*-value indicates stronger support for the clustering. In this study, we set the threshold of the AU *p*-value to 0.95. In the clustering process, the correlation was calculated by the cosine distance. This analysis identified 51 plant clusters, each with similar pharmacological effects, among 5,021 phenotypes (Supplementary Data 3).

### Extracting significantly enriched natural compounds

Significantly enriched natural compounds in plant clusters were identified by performing Fisher’s exact test. Fisher’s exact test assesses the null hypothesis of independence applying hypergeometric distribution of the numbers in a contingency table^44^. To construct the contingency table of each natural compound in the plant cluster, the number of plants was counted based on whether they were included in the cluster and whether they contain the natural compound. We performed Fisher’s exact test for each natural compound in the plant clusters with different *p*-value thresholds, including 0.1, 0.01 and 0.001 (Supplementary Table 1). The results indicated that the performance was best at 0.001, so we set the *p*-value threshold to 0.001 in this study. The average number of significantly enriched natural compounds per plant cluster was 2.4. Finally, we investigated the potential pharmacological effects of natural compounds by calculating the arithmetic mean values of the phenotype vectors of the plants belonging to the plant cluster.

### Performance evaluation

Tevaluate the performance of the proposed method, we used precision, recall, the area under the receiver operating characteristic curve (AUROC), and the area under the precision-recall curve (AUPR) defined by the following equations.

3 $$Precision=\frac{TP}{TP+FP}$$

4 $$Recall=\frac{TP}{TP+FN}$$

5 $$AUROC={\int }_{0}^{1}\frac{TP}{P}d(\frac{FP}{N})$$

6 $$AUPR={\int }_{0}^{1}\frac{TP}{TP+FP}d(\frac{TP}{P})$$

where *P*, *N*, *TP*, *TN*, *FP* and *FN* denote the numbers of real positives, real negatives, true positives, true negatives, false positives and false negatives, respectively.

## Results

### Performance evaluation

**Figure 3:**
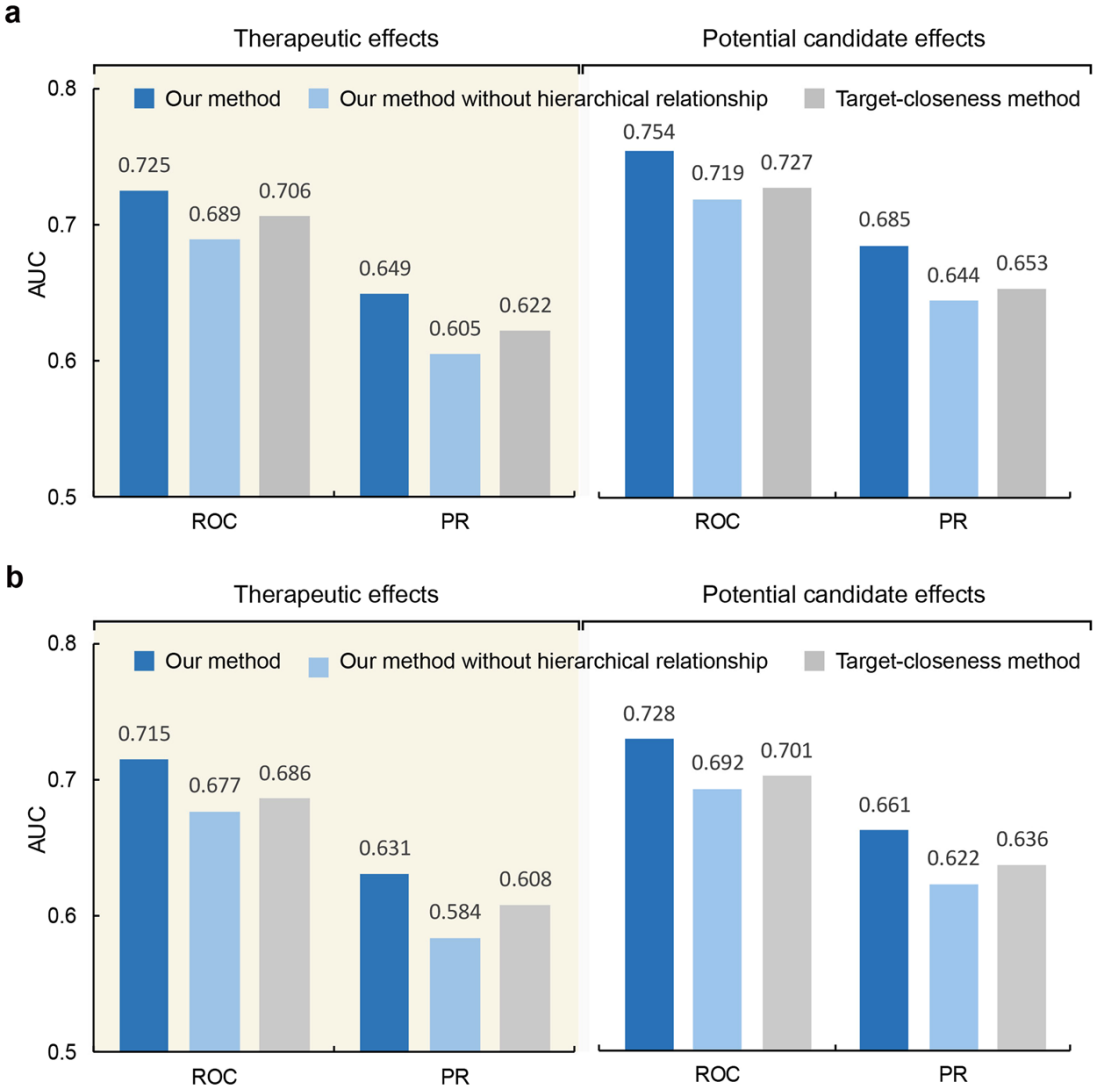
Performance evaluation of identifying pharmacological effects of natural compounds. (**a**,**b**) Average AUC scores of ROC and PR for our method (blue), our method without considering hierarchical relationships (light blue) and the target-closeness method (gray) to evaluate the performance of the prediction of pharmacological effects of natural compounds. The known therapeutic and potential candidate effects were used as gold and silver standard positive sets, respectively. Average AUC scores were calculated based on (**a**) natural compounds and (**b**) phenotypes.

Our method predicts pharmacological effects of natural compounds based on phenotype-oriented network analysis. To examine the quantitative performance of the proposed method, we calculated the average area under the curve (AUC) scores of the receiver operating characteristic (ROC) and precision-recall (PR) curves (Fig. 3). We used 21 drugs from DrugBank and 92 compounds from CTD as our gold and silver standard datasets, respectively, for assessment of the prediction of the therapeutic and potential candidate effects.

As a result, we obtained the AUROC and AUPR scores for therapeutic (AUROC_T_ = 0.725 ± 0.085, AUPR_T_ = 0.649 ± 0.080) and potential candidate effect (AUROC_P_ = 0.754 ± 0.077, AUPR_P_ = 0.685 ± 0.071) predictions. For this purpose, we averaged the AUROC and AUPR scores based on the natural compounds (Fig. 3a). To examine the importance of the hierarchical relationships, we compared the prediction performance with and without considering hierarchical relationships. The results showed that the performance decreased when hierarchical relationships were not considered in predicting therapeutic (AUROC_T_ = 0.689 ± 0.079, AUPR_T_ = 0.605 ± 0.082) and potential candidate effects (AUROC_P_ = 0.719 ± 0.060, AUPR_P_ = 0.644 ± 0.073). Furthermore, we compared our method with a network-based approach, the target-closeness method, which predicts drug efficacy by calculating the closeness between drug targets and disease genes^45^. The results indicated that our method, which uses herbal medicine information without any molecular analysis, exhibited better performance than the target-closeness method (AUROC_T_ = 0.706 ± 0.089, AUPR_T_ = 0.622 ± 0.068, AUROC_P_ = 0.727 ± 0.081, AUPR_P_ = 0.653 ± 0.062) in both therapeutic and potential candidate effect prediction. Next, we calculated the ROC and PR performance for each phenotype and averaged them to normalize the occurrence of different phenotypes (Fig. 3b) to determine whether our method focuses only on the prediction of a particular phenotype or not. For this purpose, we calculated the phenotype ranking for each natural compound based on a phenotype vector and then calculated the ROC and PR of the phenotype based on the ranking in all natural compounds. The results confirmed that when phenotype occurrence is normalized, similar performance in predicting therapeutic (AUROC = 0.715 ± 0.061, AUPR = 0.631 ± 0.064) and potential candidate effects (AUROC = 0.728 ± 0.066, AUPR = 0.661 ± 0.068) is obtained. These results indicate that our phenotype-oriented network analysis is relevant for predicting the pharmacological effects of natural compounds.

Next, by examining how the results of the proposed method differ from the results of the target-closeness method, we investigated whether it could be used as an alternative resource for drug discovery in the future. We first sorted the predicted pharmacological effects of natural compounds obtained from our method and the target-closeness method by their scores and then checked the rank correlation. The results confirmed that the rank correlation (*r*_*c*_) between the two sets was very low (*r*_*c*_ = 0.0019) and that there was no significant difference in the rank correlation scores of our results and a random set (*r*_*c*_ = 0.0012). Next, we extracted the top 10% of phenotypes from our method and from the target-closeness method and calculated the Tanimoto coefficient (*T*_*c*_) to investigate how similar the two sets were. The results confirmed that the Tanimoto coefficient between the results of our method and of the target-closeness method was very low (*T*_*c*_ = 0.065 ± 0.013) and was not significantly different from the Tanimoto coefficient between our results and a random set (*T*_*c*_ = 0.051 ± 0.010). Overall, these findings indicate that the results of our method and of the target-closeness method are statistically significantly different. The results show that the proposed method is complementary to molecular analysis and can be used as a tool to predict the pharmacological effects of natural compounds.

In contrast to the target-closeness method, which analyzes the compound efficacy via protein interaction information starting from known molecular targets, our method identifies statistically significant compounds and their efficacy by using information on the known efficacy of plants and on their constituent compounds. This discrimination provides a new way to analyze natural compounds. Previous molecular-based approaches are difficult to apply to natural compounds since the molecular target information on natural compounds is very limited (Supplementary Fig. 1). As an alternative method, we have been able to make new predictions by using the efficacy information of medicinal plants accumulated in herbal medicine and plant chemical composition information. Consequently, our method has produced novel predictions by analyzing new aspects of natural compounds.

### External literature validation

To validate the reliability of our method, we confirmed whether the predicted natural compounds and their candidate effects were identified in the external literature. We first ranked the predicted pharmacological effects of the 1,294 natural compounds by their scores and made three independent sets by selecting the top 10%, bottom 10% and random 10% of results containing 495,602 natural compound-phenotype associations. For the pharmacological effects of the selected natural compounds, we counted co-occurrences (*n*_*c*_) from PubMed abstracts, calculated the Jaccard index and conducted Fisher’s exact test (*n*_*f*_) (Table 1). We also performed the Mann-Whitney U test and calculated the corresponding *p*-values to check for significant differences in the literature evidence for the high-scored, low-scored and random sets^46^. A *p*-value from the Mann-Whitney U test lower than 0.05 was considered statistically significant.

**Table 1:** Literature validation was performed by comparing the co-occurrence, Jaccard index and Fisher’s exact test values among high-scored, low-scored and random sets. Statistical significance was calculated by the *p*-value of the Mann-Whitney U test.

		**Co-occurrence**	**Jaccard index**	**Fisher’s exact test** ^a^
High-scored (H)		1.41	2.2 × 10^−4^	3,281
Low-scored (L)		0.11	1.1 × 10^−5^	746
Random (R)		0.37	7.8 × 10^−5^	1,136
Mann-Whitney U test, *p*-value	H vs L	<0.001	<0.001	<0.001
	H vs R	<0.001	<0.001	<0.001
	L vs R	0.028	0.73	0.052

^a^*p*-value threshold of Fisher’s exact test is 0.001.

The average co-occurrence for the high-scored set (*n*_*c*_ = 1.41) was 12.8 and 3.8 times larger than the average co-occurrence of the low-scored set (*n*_*c*_ = 0.11) and the random set (*n*_*c*_ = 0.37). We also normalized the co-occurrence value by the Jaccard index to correct for the differences in the frequencies of natural compounds and of phenotypes. The average Jaccard index value of the high-scored set (JI = 2.2 × 10^−4^) was 20.0 and 2.8 times higher than the values of the low-scored set (JI = 1.1 × 10^−5^) and the random set (JI = 7.8 × 10^−5^). Furthermore, we performed Fisher’s exact test to find the significant associations (*p*-value < 0.001). To obtain the Fisher’s test value for each association, the number of PubMed abstracts that included both the natural compound and the target phenotype was counted. The number of significant associations of the high-scored set (*n*_*f*_ = 3,281) was 4.4 and 2.8 times higher than those of the low-scored set (*n*_*f*_ = 746) and the random set (*n*_*f*_ = 1,136). In addition, the *p*-values of the Mann-Whitney U test indicated that the difference in the literature evidence among the high-, low-scored and random sets was significant. These results show that our method can be used as a tool to identify pharmacological effects of natural compounds.

### Novel pharmacological effects of natural compounds

Our method uses herbal medicine information without molecular analysis to predict pharmacological effects of natural compounds, enabling us to discover effects previously undetected by the target-closeness method. To find novel pharmacological effects of natural compounds, we first filter out meaningless associations. For this purpose, we selected a cutoff for the prioritized list of phenotypes of each natural compound according to the F1-measure, the harmonic mean of precision and recall. The F1 score was calculated for the threshold of phenotype values from 0 to 0.95 with intervals of 0.05, and the best performance was obtained at 0.20. Based on this threshold value, the predicted pharmacological effects of natural compounds were filtered (Supplementary Data 4). Next, we found the PubMed evidence of predicted pharmacological effects of 10 natural compounds through manual curation (Table 2) and analyzed the results that differed from those of the target-closeness method. For instance, puerarin was investigated as a treatment for stroke^47^. However, the calculated distance between the target proteins of puerarin and the stroke-associated proteins in the molecular network shows that they are far away from each other (average shortest distance = 3.32), close to random (*p*-value < 0.001). Therefore, that the target-closeness method does not appear to show that puerarin can be used as a treatment for stroke. However, in our method, puerarin receives a high score for stroke (score = 0.773) and is proposed as a potential medicinal agent because many medicinal plants that contain tocopherol are known to be effective against stroke in herbal medicine. Furthermore, we can predict additional pharmacological effects of puerarin, such as the treatment of fever, epistaxis and perspiration, that have not been reported in DrugBank and CTD. From these results, we believe that our method can be used as an alternative tool to identify potential pharmacological effects of natural compounds.

**Table 2:** Literature evidence on the predicted pharmacological effects of natural compounds.

Compound	Phenotype	Score	Literature evidence
Puerarin	Stroke Fever	0.773 0.731	PMID: 28072733 PMID: 22401764
Berberine	Insomnia Jaundice	0.819 0.731	PMID: 28579756 PMID: 415839
Quercitrin	Amenorrhea Stomach pain	0.804 0.707	PMID: 22212502 PMID: 26758066
Spermidine	Hemorrhage Deafness Mental	0.765 0.729 0.705	PMID: 14913342 PMID: 19001365 PMID: 21501848
Choline	Anaemia Constipation Psoriasis Eczema	0.818 0.791 0.775 0.762	PMID: 15571243 PMID: 13135586 PMID: 10730754 PMID: 14896505
Genistein	Stroke Malaria	0.773 0.758	PMID: 29063799 PMID: 27585499
Eugenol	Retention of urine Urinary tract infection	0.853 0.771	PMID: 28733207 PMID: 28792229
Daidzein	Stroke	0.773	PMID: 26558782
Amentoflavone	Asthma	0.755	PMID: 27916586
Ononin	Chronic disease	0.726	PMID: 19103273

## Discussion

Herbal medicine has methodically collected information on medicinal plants for thousands of years and can be used as an important resource in drug development, in combination with information on natural compounds obtained by modern high-throughput screening techniques. Here, we introduce a phenotype-oriented network analysis to predict pharmacological effects of natural compounds from herbal medicine. The efficacy information in herbal medicine includes both high- and low-level phenotype concepts, and there are various associations between these concepts, such as synonym, symptom, superordination and subordination. Moreover, since natural sources are composed of various natural compounds, determining which natural compound is associated with a particular phenotype is difficult. In this study, the relationships between known plant efficacy and 5,021 phenotypes were quantified by considering the hierarchy of the phenotypic network. This approach enabled the extraction of plant clusters with similar efficacy by considering complex phenotype associations. From the plant clusters, we can identify significantly enriched natural compounds and their potential pharmacological effects.

The proposed method is meaningful in that pharmacological effects of natural compounds were identified by utilizing herbal medicine information, in contrast to conventional methods that focus on molecular analysis. This approach enables large-scale analysis since it can be applied even in the absence of molecular information on natural compounds. In evaluating the prediction performance, we confirmed the successful prediction of pharmacological effects of natural compounds by comparing the results with those of the target-closeness method, which relies on molecular analysis. We also found that the predicted results of the proposed method and of the target-closeness method did not overlap. This result indicates that the proposed method enabled us to identify pharmacological effects of natural compounds that went undetected by the target-closeness method.

There are some additional considerations to improve our method. First, molecular information is not taken into account in the current study. We mentioned the lack of molecular information on natural compounds as a problem of conventional methods. However, this limitation can be solved with further experiments and improved techniques. We expect more accurate predictions to be made by using both herbal medicine and molecular information appropriately. Second, advanced methods are needed to predict the pharmacological effects of natural compounds. Currently, we extract significantly enriched natural compounds from the plant cluster and map the averaged pharmacological effects of the plant cluster to the natural compounds. However, the pharmacological effects of the natural compounds and the plant cluster cannot be the same and will require further advanced analysis for precise prediction. Nevertheless, these limitations can be taken into account through further improvements. We believe that this study enables us to perform large-scale analysis and to provide a new direction for future study by systematically addressing the characteristics of herbal medicine information.

## Electronic supplementary material

Supplementary Information

Dataset 1

Dataset 2

Dataset 3

Dataset 4
