# Supplementary material for: Phenotype-oriented network analysis for discovering pharmacological effects of natural compounds
Source: Sci Rep. 2018 Aug 3;8:11667. doi: 10.1038/s41598-018-30138-w (PMC6076245; doi:10.1038/s41598-018-30138-w)
Supplement: Supplementary file 1 — Supplementary Information [file 41598_2018_30138_MOESM1_ESM.docx]

**Supplementary information for**

**Phenotype-oriented network analysis for discovering pharmacological effects of natural compounds**

Sunyong Yoo^1,2^, Hojung Nam^3*^ and Doheon Lee^1,2*^

^1^Department of Bio and Brain Engineering, Korea Advanced Institute of Science and Technology (KAIST), Daejeon 34141, Republic of Korea

^2^Bio-Synergy Research Center, Daejeon 34141, Republic of Korea

^3^School of Electrical Engineering and Computer Science, Gwangju Institute of Science and Technology (GIST), Gwangju 61005, Republic of Korea

**Inventory**

Supplementary Figures

Supplementary Tables

**Supplementary Figures**

**Supplementary Figure 1. Comparing known molecular structures and targets between drugs and natural compounds. (a)** Comparison of known molecular structure information on drugs and natural compounds. While 87% of drugs (left circle graph) have molecular structure information, only 45% of natural compounds (right circle graph) have molecular structure information. **(b)** Comparison of known protein targets between drugs and natural compounds. While 86% of drugs (left circle graph) have protein target information, only 3% of natural compounds (right circle graph) have protein target information.


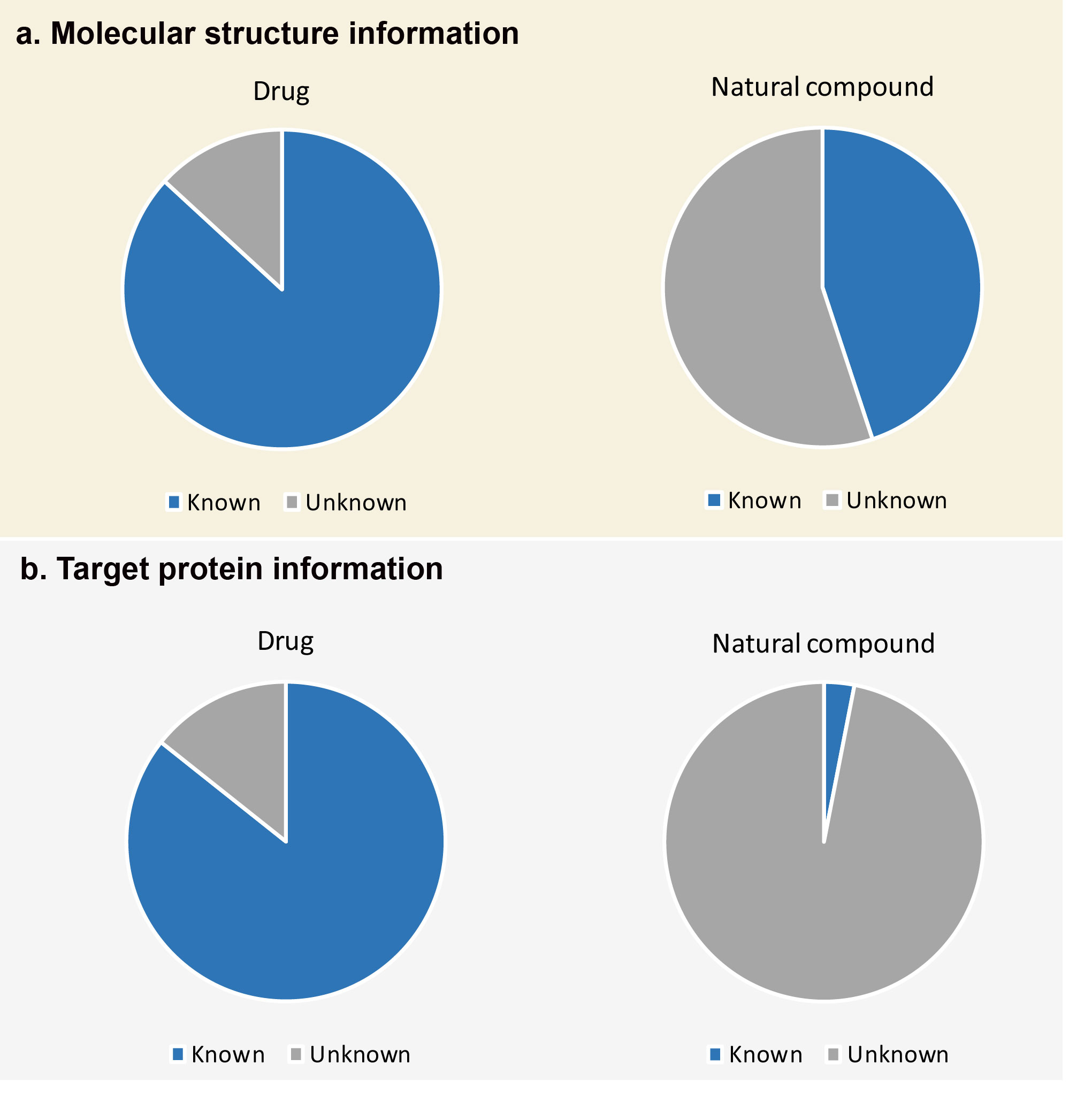

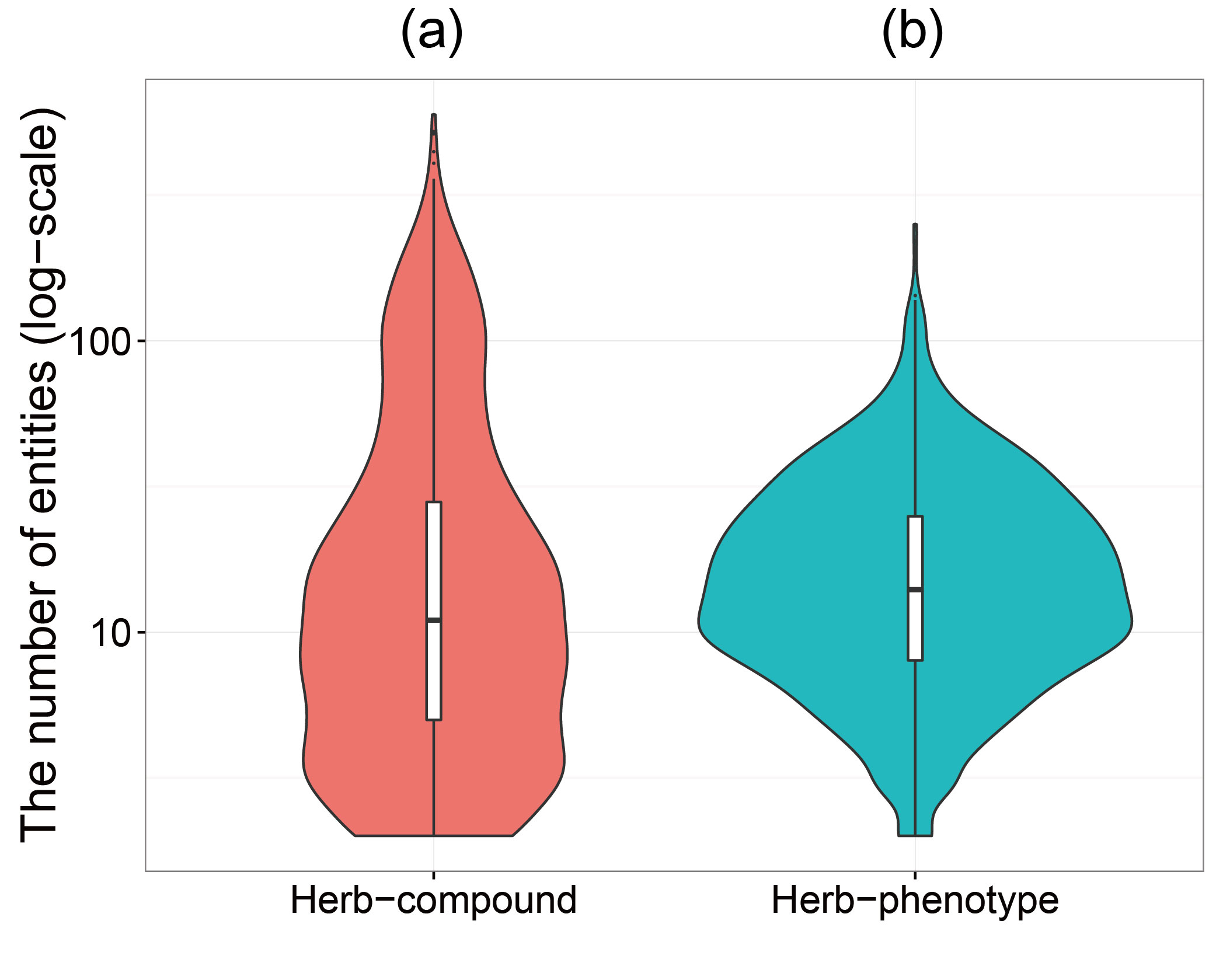


**Supplementary Figure 2. Distribution of the number of compounds contained in herbs and the phenotypes associated with herbs. (a)** Distribution of the number of compounds per herb. The mean number of compounds per herb is 19.9. **(b)** Distribution of the number of phenotypes per herb. The mean number of phenotypes per herb is 31.8.


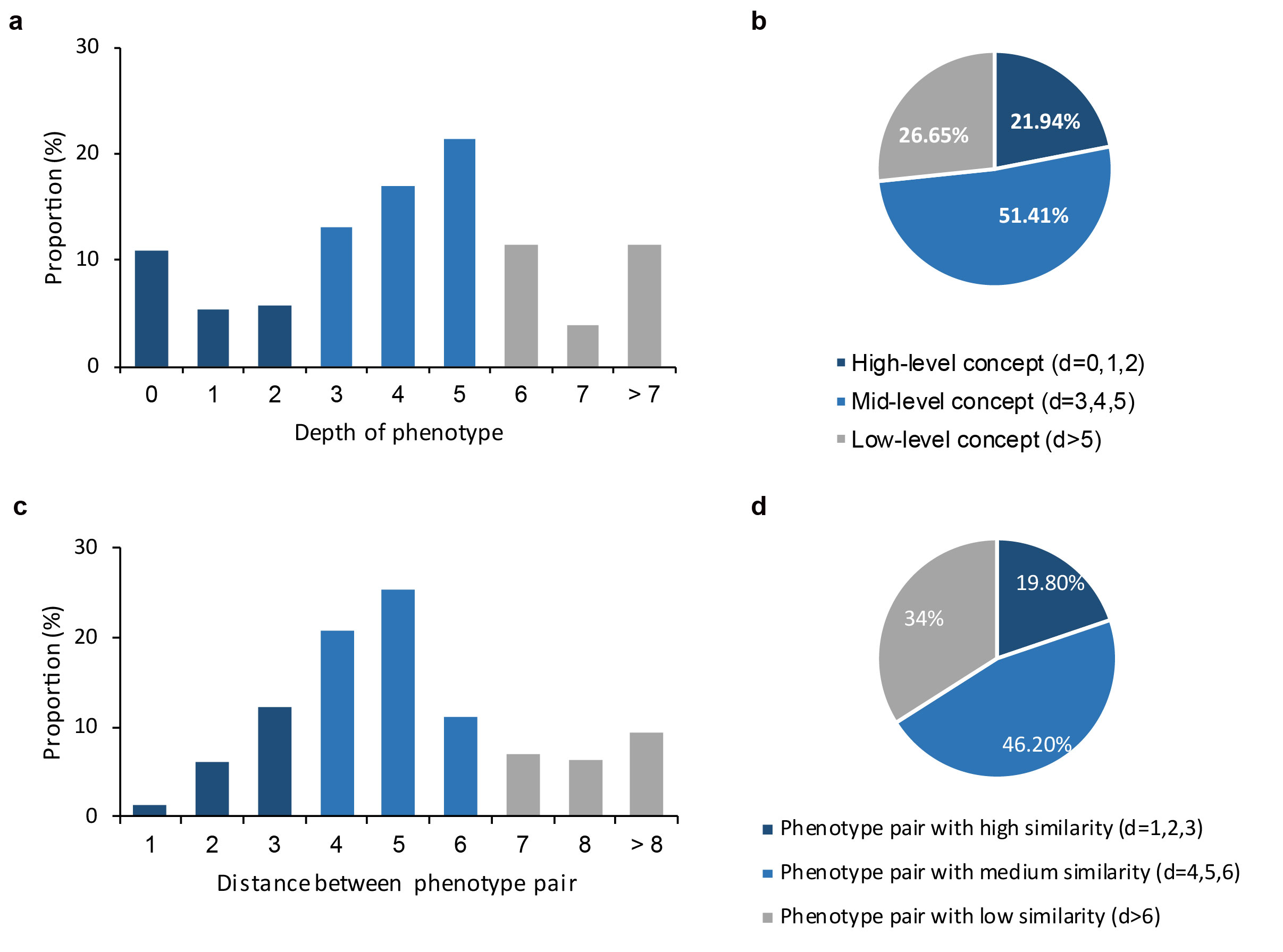


**Supplementary Figure 3. Characteristics of herbal medicine. (a)** Histogram of the proportion of phenotypes by depth. Known phenotypes of each herb were mapped to the phenotypic network, and the phenotype depth was found by calculating the distance from the root to the phenotype. The average proportion was calculated based on the herb. **(b)** The proportion of phenotypes for high-level, mid-level and low-level concepts. **(c)** Histogram of the proportion of the distance of a phenotype pair. **(d)** The proportions of phenotype pairs for high, medium and low similarity.

**Supplementary Tables**

**Supplementary Table 1. The relationship between ROC and PR performance according to *p*-value threshold.**

| *p*-value | Therapeutic effects | | Potential candidate effects | |
| --- | --- | --- | --- | --- |
|  | ROC | PR | ROC | PR |
| 0.1 | 0.648±0.058 | 0.607±0.043 | 0.679±0.045 | 0.624±0.044 |
| 0.01 | 0.691±0.071 | 0.622±0.055 | 0.717±0.058 | 0.643±0.054 |
| 0.001 | 0.725±0.085 | 0.649±0.080 | 0.754±0.077 | 0.685±0.071 |
